# Supplementary material for: Teaching-learning in clinical education based on epistemological orientations: A multi-method study
Source: PLoS One. 2023 Nov 30;18(11):e0289150. doi: 10.1371/journal.pone.0289150 (PMC10688630; doi:10.1371/journal.pone.0289150)
Supplement: S1 File — (DOCX) [file pone.0289150.s001.docx]

**“In the Name of God”**

**Remote qualitative interviews (web-based and telephone)**

- **Dear participant, greetings, and regards**

The following questions are for conducting the second sub-study of Shahid Beheshti University of Medical Sciences' doctoral dissertation on medical education, title "Representation of the teaching-learning in the clinical education of general medicine in Iran, with an emphasis on its challenges" using situational analysis method as a post-structural version of grounded theory. These open-ended questions are solely for scientific research, and your responses will be kept confidential. Please read the questions carefully and patiently help us to complete them and achieve my research goals.

Thank you in advance for your participation.

- Please introduce yourself {such as demographic information; gender, age, academic semester (if you are a student), and teaching experience or educational activities (if you are a teacher)}

**Response --------------------------------------------------------------------------------------------------------**

1- What are the components and elements (human, non-human, material, symbolic, and discursive) of teaching-learning in clinical education of general medicine in Iran? (You can think broadly in answering this question, and you are completely free to express these components)

**Response --------------------------------------------------------------------------------------------------------**

2- Explain the importance of each of these elements and components about this specific situation (teaching-learning in the clinical education of general medicine in Iran).

**Response --------------------------------------------------------------------------------------------------------**

3- What are the arenas of teaching-learning in the clinical education of general medicine in Iran? Who or which social worlds (social beings) are present in these arenas?

**Response --------------------------------------------------------------------------------------------------------**

4- What components and elements in the teaching-learning process in the clinical education of general medicine in Iran lead to the facilitation of students' learning and their achievement of learning goals and outcomes?

List the items in order of importance and explain your point of view.

**Response --------------------------------------------------------------------------------------------------------**

5- What are the challenges of clinical education in general medicine in Iran (concerning teaching-learning)?

Explain your point of view.

**Response --------------------------------------------------------------------------------------------------------**
