# Supplementary material for: Teaching-learning in clinical education based on epistemological orientations: A multi-method study
Source: PLoS One. 2023 Nov 30;18(11):e0289150. doi: 10.1371/journal.pone.0289150 (PMC10688630; doi:10.1371/journal.pone.0289150)
Supplement: S2 File — (DOCX) [file pone.0289150.s002.docx]

**“In the Name of God”**

**Expert panel questions (question-centered and discussion phase)**

**Questions before forming the panel of experts**

In the third sub-study, to collect data before forming a group discussion in the expert panel, the following two questions were sent to experts via Porsline (web-based):

1. How do you define effective teaching-learning in clinical education?
2. What components and elements should be included in clinical teaching-learning in undergraduate medical education to enable students to achieve the goals and outcomes of clinical learning?

**Expert panel's discussion**

In the first round of this panel, the first and second sub-studies (purpose, design, and findings) were fully presented and explained to the members, the participants' questions were answered, and a group discussion was conducted.

Then, in the second round, the initial model, which was the result of the synthesis of the findings of the first and second sub-studies, which was obtained in a deductive manner and based on the synthesis of the framework, was provided to the members. Based on the following three questions, the model was discussed and developed:

1. What do you think about this model?
2. Is the developed model comprehensive, and can its dimensions and components lead to the achievement of learning outcomes in clinical education?
3. What other dimensions or components could there be that have been neglected in this model?
