# Supplementary material for: Teaching-learning in clinical education based on epistemological orientations: A multi-method study
Source: PLoS One. 2023 Nov 30;18(11):e0289150. doi: 10.1371/journal.pone.0289150 (PMC10688630; doi:10.1371/journal.pone.0289150)
Supplement: S3 File — (DOCX) [file pone.0289150.s003.docx]

**“In the Name of God”**

**Open-ended questions related to epistemological orientations toward teaching and learning**

In the final phase of the third sub-study, based on the literature, eight open-ended questions regarding the epistemological orientations of experts about teaching and learning were developed to construct a framework for synthesis based on the best fit. The questions are as follows:

1. What is teaching? Or what does it mean to teach?

2. What aims or objectives do you pursue in your teaching?

3. How do you see your role and responsibility, students, patients, and others (such as nurses, residents, hospital physicians, attending physicians, and other medical personnel) in the educational arenas in teaching and learning process?

4. How do you define effective teaching? What are the barriers to effective teaching?

5. What is learning? Or what does it mean to learn?

6. Can the student take responsibility for his or her learning? How?

7. As a teacher, does your teaching influence students' learning? How?

8: How does student learning influence your teaching?
