# Supplementary material for: Teaching-learning in clinical education based on epistemological orientations: A multi-method study
Source: PLoS One. 2023 Nov 30;18(11):e0289150. doi: 10.1371/journal.pone.0289150 (PMC10688630; doi:10.1371/journal.pone.0289150)

PLOS ONE

Teaching-learning in clinical education Based on epistemological orientations: A multi-method study

--Manuscript Draft--

**Manuscript Number:**	PONE-D-23-10842
**Article Type:**	Research Article
**Full Title:**	Teaching-learning in clinical education Based on epistemological orientations: A multi-method study
**Short Title:**	Teaching-learning in Medical education
**Corresponding Author:**	Hamed Khani, Ph.D.<br>Shaheed Beheshti University of Medical Sciences: Shahid Beheshti University of Medical Sciences<br>Tehran, IRAN, ISLAMIC REPUBLIC OF
**Keywords:**	Medical Education; teaching-learning; epistemological orientation; triangulation; qualitative inquiry
**Abstract:**	Introduction: Teaching-learning is the heart of medical education in the clinical setting. The objective of this research was to develop a conceptual model of effective clinical teaching in undergraduate medical education and conceptualize its operational framework based on the best fit approach.<br>Materials and Methods: This research consisted of three sub-studies conducted using a multi-method approach. The first sub-study was conducted using a qualitative meta-synthesis approach. The second sub-study used Clarke's situational analysis approach as a postmodern version of grounded theory. Finally, the third sub-study was designed in two stages. First, it was conducted using the expert panel, in the second step, framework of synthesis based on best fit, and the framework of Ottenhoff- de Jonge et al., which formed the basis of this study.<br>Results: In the first sub-study, qualitative evidence on the factors of effective teaching-learning in clinical education was synthesized into five dimensions. Based on the second sub-study, the clinical teaching-learning situation in undergraduate medical education in Iran was represented in three maps, including situational, social worlds/arenas, and positional. Finally, in the third sub-study, based on model modification and development in the expert panel, the effective teaching-learning dimensions were developed into behavioral, social, pedagogical, technology, contextual, educational leadership, and financial dimensions. In the second step, based on the framework of Ottenhoff- de Jonge et al., a three-dimensional matrix was developed concerning epistemological orientations about teaching and learning.<br>Discussion: Moving from a single teaching-centered and learning-centered orientation to a teaching-learning-centered orientation is required for effective teaching-learning in clinical medical education.
**Order of Authors:**	Hamed Khani, Ph.D.
	Soleiman Ahmady
	Babak Sabet
	Shirdel Zandi
	Mojgan Mohammadimehr
	Somayeh Niakan
**Additional Information:**	
**Question**	**Response**
**Financial Disclosure**<br><br>Enter a financial disclosure statement that describes the sources of funding for the	The authors received no specific funding for this work.

work included in this submission. Review the submission guidelines for detailed requirements. View published research articles from *PLOS ONE* for specific examples.<br><br>This statement is required for submission and **will appear in the published article** if the submission is accepted. Please make sure it is accurate.<br><br>**Unfunded studies**<br>Enter: *The author(s) received no specific funding for this work.*<br><br>**Funded studies**<br>Enter a statement with the following details:<br>• Initials of the authors who received each award<br>• Grant numbers awarded to each author<br>• The full name of each funder<br>• URL of each funder website<br>• Did the sponsors or funders play any role in the study design, data collection and analysis, decision to publish, or preparation of the manuscript?<br>• **NO** - Include this sentence at the end of your statement: *The funders had no role in study design, data collection and analysis, decision to publish, or preparation of the manuscript.*<br>• **YES** - Specify the role(s) played.<br><br>* typeset	
**Competing Interests**<br><br>Use the instructions below to enter a competing interest statement for this submission. On behalf of all authors, disclose any competing interests that could be perceived to bias this work—acknowledging all financial support and any other relevant financial or non-financial competing interests.<br><br>This statement is required for submission and **will appear in the published article** if the submission is accepted. Please make sure it is accurate and that any funding sources listed in your Funding Information	The authors have declared that no competing interests exist.

Powered by Editorial Manager® and ProduXion Manager® from Aries Systems Corporation

later in the submission form are also declared in your Financial Disclosure statement.<br><br>View published research articles from *PLOS ONE* for specific examples.<br><br>**NO authors have competing interests**<br><br>Enter: *The authors have declared that no competing interests exist.*<br><br>**Authors with competing interests**<br><br>Enter competing interest details beginning with this statement:<br><br>*I have read the journal's policy and the authors of this manuscript have the following competing interests: [insert competing interests here]*<br><br>* typeset	
**Ethics Statement**<br><br>Enter an ethics statement for this submission. This statement is required if the study involved:<br><br>• Human participants<br>• Human specimens or tissue<br>• Vertebrate animals or cephalopods<br>• Vertebrate embryos or tissues<br>• Field research<br><br>Write "N/A" if the submission does not require an ethics statement.<br><br>General guidance is provided below. Consult the submission guidelines for detailed instructions. **Make sure that all information entered here is included in the Methods section of the manuscript.**	Ethical approval and consent to participate<br>This article is taken from the Ph.D. dissertation of Dr. Hamed Khani from the Department of Medical Education of Shahid Beheshti University of Medical Sciences and has received ethics approval with the number IR.SBMU.SME.REC.1399.097 on 2021-01-13 from the university’s ethics committee. Verbal and written consent was obtained from all participants to participate in telephone interviews (voice recording), web-based interviews, and expert panel. All participants were informed of the research objectives at the time of data collection and were assured that participation in qualitative interviews (web-based and telephone) and expert panel was voluntary. Data confidentiality and anonymity of participants were guaranteed in the qualitative interviews, and expert panel, both verbally and writing. Finally, all participants were informed that will be presented to them the research results if they request. All methods were conducted in accordance with the ethical principles of the Declaration of Helsinki.

Powered by Editorial Manager® and ProduXion Manager® from Aries Systems Corporation

**Format for specific study types**<br><br>**Human Subject Research (involving human participants and/or tissue)**<br>• Give the name of the institutional review board or ethics committee that approved the study<br>• Include the approval number and/or a statement indicating approval of this research<br>• Indicate the form of consent obtained (written/oral) or the reason that consent was not obtained (e.g. the data were analyzed anonymously)<br><br>**Animal Research (involving vertebrate animals, embryos or tissues)**<br>• Provide the name of the Institutional Animal Care and Use Committee (IACUC) or other relevant ethics board that reviewed the study protocol, and indicate whether they approved this research or granted a formal waiver of ethical approval<br>• Include an approval number if one was obtained<br>• If the study involved *non-human primates*, add *additional details* about animal welfare and steps taken to ameliorate suffering<br>• If anesthesia, euthanasia, or any kind of animal sacrifice is part of the study, include briefly which substances and/or methods were applied<br><br>**Field Research**<br><br>Include the following details if this study involves the collection of plant, animal, or other materials from a natural setting:<br>• Field permit number<br>• Name of the institution or relevant body that granted permission	
**Data Availability**<br><br>Authors are required to make all data underlying the findings described fully available, without restriction, and from the time of publication. PLOS allows rare exceptions to address legal and ethical concerns. See the PLOS Data Policy and FAQ for detailed information.	Yes - all data are fully available without restriction

Powered by Editorial Manager® and ProduXion Manager® from Aries Systems Corporation

A Data Availability Statement describing where the data can be found is required at submission. Your answers to this question constitute the Data Availability Statement and **will be published in the article**, if accepted.<br><br>**Important:** Stating 'data available on request from the author' is not sufficient. If your data are only available upon request, select 'No' for the first question and explain your exceptional situation in the text box.<br><br>Do the authors confirm that all data underlying the findings described in their manuscript are fully available without restriction?	
**Describe where the data may be found in full sentences. If you are copying our sample text, replace any instances of XXX with the appropriate details.**<br><br>• If the data are **held or will be held in a public repository**, include URLs, accession numbers or DOIs. If this information will only be available after acceptance, indicate this by ticking the box below. For example: *All XXX files are available from the XXX database (accession number(s) XXX, XXX.).*<br>• If the data are all contained **within the manuscript and/or Supporting Information files**, enter the following: *All relevant data are within the manuscript and its Supporting Information files.*<br>• If neither of these applies but you are able to provide **details of access elsewhere**, with or without limitations, please do so. For example:<br><br>*Data cannot be shared publicly because of [XXX]. Data are available from the XXX Institutional Data Access / Ethics Committee (contact via XXX) for researchers who meet the criteria for access to confidential data.*<br><br>*The data underlying the results presented in the study are available from (include the name of the third party*	Data and materials availability statement<br>The data that support the findings of this study are not publicly available, but can be available from the authors on reasonable request. All datasets generated and analyzed and other materials during the current study (such as; qualitative interviews questions; expert panel questions/question-centered and discussion phase; and open-ended questions related to epistemological orientation towards teaching and learning) are available from the corresponding author on reasonable request.

and contact information or URL).<br>• This text is appropriate if the data are owned by a third party and authors do not have permission to share the data.<br><br>* typeset	
Additional data availability information:	

Manuscript Click here to access/download;Manuscript;Manuscript.docx

Full title:

Teaching-learning in clinical education Based on epistemological orientations: A multi-method study

Short title:

Teaching-learning in Medical education

Hamed Khani[1*], Soleiman Ahmady[1], Babak Sabet[2], Shirdel Zandi[3], Mojgan Mohammadimehr[4] and Somayeh Niakan[5]

[1]Department of Medical Education, Virtual School of Medical Education & Management, Shahid Beheshti University of Medical Sciences, Tehran, Iran.

[*]Department of Medical Education, Virtual School of Medical Education & Management, Shahid Beheshti University of Medical Sciences, Tehran, Iran. (Corresponding author) Tel: +98-9184528354, Email: hkhani95@sbmu.ac.ir, hkhani83@yahoo.com

[2]Department of Surgery, School of Medicine, Shahid Beheshti University of Medical Sciences, Tehran, Iran.

[3]Department of Operating Room, School of Paramedicine, Hamadan University of Medical Sciences, Hamadan, Iran.

[4]Department of Laboratory Sciences, School of Paramedicine, Aja University of Medical Sciences, Tehran, Iran.

[5]Department of Prosthodontics, School of Dentistry, Tehran University of Medical Sciences. Tehran, Iran

Abstract

Introduction: Teaching-learning is the heart of medical education in the clinical setting. The objective of this research was to develop a conceptual model of effective clinical teaching in undergraduate medical education and conceptualize its operational framework based on the best fit approach.

Materials and Methods: This research consisted of three sub-studies conducted using a multi-method approach. The first sub-study was conducted using a qualitative meta-synthesis approach. The second sub-study used Clarke's situational analysis approach as a postmodern version of grounded theory. Finally, the third sub-study was designed in two stages. First, it was conducted using the expert panel, in the second step, framework of synthesis based on best fit, and the framework of Ottenhoff- de Jonge et al., which formed the basis of this study.

Results: In the first sub-study, qualitative evidence on the factors of effective teaching-learning in clinical education was synthesized into five dimensions. Based on the second sub-study, the clinical teaching-learning situation in undergraduate medical education in Iran was represented in three maps, including situational, social worlds/arenas, and positional. Finally, in the third sub-study, based on model modification and development in the expert panel, the effective teaching-learning dimensions were developed into behavioral, social, pedagogical, technology, contextual, educational leadership, and financial dimensions. In the second step, based on the framework of Ottenhoff- de Jonge et al., a three-dimensional matrix was developed concerning epistemological orientations about teaching and learning.

Discussion: Moving from a single teaching-centered and learning-centered orientation to a teaching-learning-centered orientation is required for effective teaching-learning in clinical medical education.

Keywords: Medical education; teaching-learning; epistemological orientation; triangulation; qualitative inquiry

Introduction

The clinical environment can be defined as a situation with the presence of a medical educator, medical students, clinical staff, and patients revolving around the diagnosis, treatment, and care of the patients and teaching-learning activities. This includes two important points: First, the clinical environment consists of inpatient, outpatient, and community settings [1]. Second, teaching in clinical settings is often done in routine clinical care where patients and their problems are the basis of teaching to medical students [2]. A wide range of professional skills such as communication skills, professionalism, history taking, and physical examination required for medical practice are taught in these environments and settings. But what matters is the effectiveness of teaching and students' achievement of clinical learning outcomes. In other words, clinical teaching-learning should incorporate the components and characteristics that contribute to students achieving learning outcomes.

Achieving effective teaching in any educational environment requires the formation of an efficient and high-quality teaching-learning process or system.

Higher education teaching-learning consists of various components and aspects that can be analyzed holistically within the framework of an efficient behavioral system. As a system model, these components and dimensions can be used to create the teaching-learning system in the context of a strategic approach and with appropriate leadership [3].

Comprehensive studies have not been conducted on the dimensions of effective teaching in clinical education. For example, a study focused on successful clinical education and considered components such as the tutor's role, the student's role, the patient's roles and characteristics, and the characteristics of a good clinical environment [4]. Moreover, the perceptions of clinical teachers and students of effective opportunities to facilitate learning in a clinical context have been considered [5]. In another study, Ross & Stenfors-Hayes [6] developed a framework for teaching and learning. This framework includes teacher and

teaching activities, learner and learning activities, shared activities between teachers and learners, teaching and learning situations, and content. They propose this framework for the undergraduate course and do not consider the distinction between preclinical and clinical contexts.

In addition, in recent decades, studies have been published that have conceptualized teaching and learning in education, higher education, and medical education based on a framework [3, 7-12].

This line of research is significant. Because it has implications for teaching and learning activities and contributes to teaching-learning theory, in other words, classifications related to the orientation of teaching-learning are essential and determine the direction of the teaching-learning system. By focusing on its dimensions, the quality of education can be improved. However, these categorizations are limited to teaching-learning in medical education, especially clinical education. Therefore, this study aimed at conceptualizing effective clinical teaching-learning in undergraduate medical education based on epistemological orientations about teaching-learning. Therefore, the objective of this research was to develop a conceptual model of effective clinical teaching in undergraduate medical education and conceptualize its operational framework based on the best fit approach.

Materials and methods

This study comprised three sub-studies conducted using a multi-method approach (Data and methods triangulation). This research data were collected through multiple methods such as synthesis of qualitative studies, literature review, analysis of the general medicine curriculum and related documents, remote semi-structured interviews (web-based and telephone), expert panel, and the framework synthesis based on the best fit. In addition to documents and curriculum analysis, experienced clinical teachers, medical education specialists, and eligible medical students participated in this study. This study lasted from January 2021 to April 2022.

First sub-study: Systematic review of qualitative studies and meta-synthesis

The purpose of the first sub-study was to develop a comprehensive framework of effective teaching-learning factors in clinical education. This sub-study was performed based on qualitative meta-synthesis and the seven-step method of Sandelowski & Barroso [13]. The studies' identification, screening, and selection were performed according to the PRISMA[1] flowchart instructions. PICOS[2] strategy was used to formulate the research question and determine the criteria for eligible studies. The determined electronic databases and journals were searched from 1990 to 2021 to identify related studies and articles. In order to increase the reliability of the search, this process was performed by two researchers, one of whom was a librarian. Based on the composition and search strategies in the databases (OVID, PubMed, Web of Science, SCOPUS, Eric, Magiran, and SID), 33,799 and 56 studies were identified from other sources. After removing duplicates, 29,285 studies and articles remained. Study screening was performed based on inclusion and exclusion criteria by two authors. After initial screening and assessment based on the abstract, 120 studies and articles remained. The eligibility assessment (full-text assessment) was conducted by two researchers simultaneously and independently. At this step, any disagreement between the researchers was discussed and resolved. In the event of significant inconsistencies, we sought assistance from other research team members or an expert outside the research team. After monitoring and reviewing the full text of the publications, 53 studies and articles were selected, and 45 studies and articles were chosen and included in the meta-synthesis process after a critical assessment of candidate studies using the CASP tool. In order to analyze and synthesize the findings of qualitative studies, inductive coding (reading and reading carefully studies, open, axial and selective coding and production of analytical themes) were used. To ensure the data's trustworthiness, Maxwell [14, 15] criteria, such as descriptive, interpretative, and theoretical validity, as well as Kvale [16] criteria, such as pragmatic validity, were considered in this sub-study.

Second sub-study: The Situational analysis of teaching-learning in clinical education

Clarke's situational analysis approach [17] was used as a post-structural version of grounded theory in this sub-study. The purpose of this sub-study was to represent the fundamental elements and components of clinical teaching-learning in undergraduate medical education in Iran, focusing on identifying the challenges of effective teaching-learning. The data of this sub-study were collected using several methods and sources such as a mini literature review, an analysis of the general medicine curriculum and related documents, and remote qualitative interviews (web-based and telephone). The participants of this sub-study were purposefully selected and entered the research process through the methods of maximum variation, snowball (experienced clinical teachers, medical education specialists and students) and convenience sampling (documents and curriculum analysis). In this sub-study, thirty-one people (including experienced clinical teachers, medical education specialists and students) responded to the web-based interviews out of the invited forty. Also, seven people (including experienced clinical teachers and medical education specialists) participated in the telephone interviews. The interview questions included an introductory question to establish rapport with the interviewees and five main open-ended questions. Web-based and telephone interviews questions were the same, with the difference that in the telephone interviews additional questions were developed during the conversations. However, many medical educators, clinical teachers, and students received web-based interview questions via Porsline and answered only the questions included. The web-based interviews were used to acquire a broad understanding of the situation, followed by the telephone interviews (video and voice call) to gain a rich and deep insight into the people involved.

The average time to answer qualitative interviews (web-based and telephone) lasted about 30 minutes (between 15 to 40 minutes). The notes of the documents, curriculum analysis, and the transcripts of the qualitative interviews were analyzed for the emergence and categorization of

sub-themes and themes. Finally, the themes from all data sources were combined to form a comprehensive picture of the situation, represented in three maps. The researchers used three maps (situational, social worlds/arenas, and positional) as the main strategies for situational analysis throughout the research project (from design to reporting). Lincoln and Guba's criteria [18] were used to increase the rigor of the data in this study. Memoing, prolonged engagement with data, member checking, peer checking, coding and categorization of the emerging themes by the researchers, and establishing a consensus were all employed to assure the credibility and dependability of the findings. To guarantee the confirmability of strategies such as devoting sufficient time to data collection and analysis, utmost accuracy in the research process and audit trail were used. Finally, to ensure the transferability of strategies such as the thick description of the results in the form of discussion about the findings, quality assessment of data by two medical education specialists and experienced clinical teachers and different participants in terms of position were used.

Third sub-study: Expert Panel and Best fit approach

The purpose of this sub-study was to present a conceptual model of effective teaching in clinical education and conceptualize its operational framework based on epistemological orientations about teaching and learning. This sub-study was conducted using a qualitative approach (expert panel method and best-fit approach).

Expert panel method

The expert panel method is the forum in which prominent and expert people are invited to express and share their experiences, thoughts, and ideas' in a particular field [19]. This method is often based on the modified Delphi structure [20].

In this sub-study, an expert panel was used, which included two phases question-centered and discussion. In the first step, in order to collect data, before forming a group discussion in the expert panel, the following two questions were sent to 12 experts via Porsline (web-based):

How do you define effective teaching-learning in clinical education?

What components and elements should be included in clinical teaching-learning in undergraduate medical education to enable students achieve the goals and outcomes of clinical learning?

After collecting the responses to these questions, and implementing and assessing them, a 10-member expert panel was constituted (6 medical education specialists and four clinical teachers). This panel was held virtually through the Skype platform in two rounds (Each round in 2 hours). In addition to these two rounds, other data were collected through the WhatsApp group in an unstructured manner. In the first round, a brief introduction was given, and the members' and research team's expectations were stated to develop rapport between the person in charge of the panel and the members and between members. The head of the panel (the research team's leader) then presented and discussed the first and second sub-studies (purpose, design, and findings) to the members, answering questions and leading a group discussion. In the next step, the second round of the expert panel was held with an interval of one week and focused on developing and modifying the model obtained from the first sub-study based on the components and elements obtained from the situational analysis (second sub-study). At this stage, the interview guide (discussion) was used. However, before the second round and with an interval of one week between the first and second rounds, the findings and results of both sub-studies were shared in a schematic to facilitate a logical and objective consensus among the panel members form in the WhatsApp group. Eight open-ended questions were sent privately over WhatsApp in order to frame synthesis, and participants were requested to return the responses in the form of a recorded voice. This aimed to achieve an overview of their epistemological orientation in relation to teaching and learning. The participants of this sub-study entered the research process purposefully through reputational case sampling. The data from this sub-study was recorded, implemented, and coded at each step, and sub-categories and categories were classified using direction content analysis. The rigor and trustworthiness

of the data in this sub-study depended entirely on guaranteeing the trustworthiness of the data in the first and second sub-studies. In addition, the final model was examined and validated in this step by the supervisor and expert peer review (two external experts who are specialists in the field) and their feedback was used.

Framework synthesis based on the best fit

Framework synthesis is one of the methods developed for synthesizing qualitative data, which is mainly a deductive approach [21]. The "best fit" framework synthesis method is an approach and mean for testing, reinforcing, and developing an existing published model or framework, which is presented for a potentially different but relevant population (same context) [22]. Framework synthesis based on best fit requires identifying a framework, theory, or conceptual model related to the research subject. Following steps such as a systematic review of qualitative studies and meta-synthesis (first sub-study), situational analysis (second sub-study), and model development based on the first and second sub-studies using an expert panel, which was somewhat in line with the method proposed by Carroll et al., [23] the authors focused on reviewing existing published frameworks and models of teaching-learning in the fields of education, higher education, and medical education in this phase. The framework synthesis approach was used after they discovered a published framework in the literature that conceptualized teaching-learning in the practice of medical education [12]. Although this framework did not fully match the research subject, it had the best fit. Finally, an operational framework was conceptualized based on the analysis of participants' responses to open-ended questions in relation to their beliefs about teaching and learning and using the best fit approach. Two sets of inclusion criteria and a search and selection of studies and articles are required in the best-fit approach. The first is for a systematic review of qualitative studies conducted in the first sub-study, and the second is for searching and identifying a related model or framework. These criteria, which are consistent with the PICO strategy (population, intervention,

comparison, outcome) and the SPICE strategy (setting, perspective, intervention/phenomena of interest, comparison, and evaluation), are presented in Table (1) for both systematic reviews of qualitative studies and the identification of related model and framework. The reliability of the search was ensured by utilizing these two strategies.

Table 1. Inclusion criteria for systematic review of qualitative studies and identification of related framework

→ ↓	**Models/ frameworks and theories**	**Primary qualitative studies (first sub-study)**
**Setting/ population**	Undergraduate Medical Education/ students/ educators / patients	Clinical education in undergraduate medical/ Clinical training in undergraduate medical/ students / educators / patients
**Intervention/ phenomena of interest**	Teaching-learning in higher education and medical education	Effective teaching-learning in clinical education
**Research design/ evaluation**	Existing published frameworks and models on teaching-learning in medical education	Qualitative approach, grounded theory, ethnography, phenomenology, thematic analysis, content analysis, Delphi method, focus groups and discourse analysis

Frameworks of belief orientations about teaching and learning

A number of studies have classified the belief orientation about teaching and learning on a continuum from teaching-centered to learning-centered [7, 24, 25, 8, 9, 10, 11, 3, 12]. One of these frameworks proposed for the context of medical education is the framework of Ottenhoff-de Jonge et al., [12]. They developed a new framework for the context of medical education based on the Samuelowicz and Bain framework [10], which focused on medical educators' beliefs about teaching, learning, and knowledge. Their proposed framework is comprised of a two-dimensional matrix in belief orientations (including six belief orientations: imparting information, transmitting structured knowledge, facilitating understanding, helping the student develop expertise, sharing the responsibility for developing expertise, and negotiating meaning) and nine belief dimensions (including desired learning outcomes, expected use of knowledge, responsibility for transforming knowledge, nature of knowledge, students' existing

conceptions, teacher-student interaction, creation of a conducive educational environment, professional development and student motivation). We selected this framework for the following three reasons. First, this framework was largely matched with the subject of our research and therefore had the best fit. Second, this framework is the most comprehensive of all the frameworks developed in the literature. Third, this framework has been developed for the context of medical education.

Ethical approval and consent to participate

This article is taken from the Ph.D. dissertation of Dr. Hamed Khani from the Department of Medical Education of Shahid Beheshti University of Medical Sciences and has received ethics approval with the number IR.SBMU.SME.REC.1399.097 on 2021-01-13 from the university's ethics committee. Verbal and written consent was obtained from all participants to participate in telephone interviews (voice recording), web-based interviews, and expert panel. All participants were informed of the research objectives at the time of data collection and were assured that participation in qualitative interviews (web-based and telephone) and expert panel was voluntary. Data confidentiality and anonymity of participants were guaranteed in the qualitative interviews, and expert panel, both verbally and writing. Finally, all participants were informed that will be presented to them the research results if they request. All methods were conducted in accordance with the ethical principles of the Declaration of Helsinki.

Results

Findings of first sub-study

According to the findings of the first sub-study, seven components from the included qualitative research were synthesized into five primary dimensions, and a useful teaching-learning framework for clinical education was developed. This framework includes dimensions such as behavioral or content (learner, teacher, patient and her/his behavior), social (collaborative learning community), pedagogical (instructional design and teaching-learning

opportunities), the context of teaching-learning (positive and supportive clinical environment) and educational leadership (classroom management and structure) (see Figure 1). [26].

Figure 1. Results of qualitative meta-synthesis in relation to the components and dimensions of effective teaching-learning in clinical education

Findings of second sub-study

Based on the results of this sub-study, clinical teaching-learning in undergraduate medical education in Iran was represented in three maps. The first map formulated human, non-human, material, symbolic and discursive components and elements for teaching-learning. Then, the discourses and themes that emerged in the messy version were organized and specifically represented using the ordered version of the situational map. The second map represented social worlds/arenas of teaching-learning situations in the clinical training in undergraduate medical education in Iran. According to this map, the three main arenas such as a hospital (including ambulatory education and training in the outpatient clinics, education in hospital wards, inpatient settings, bedside teaching and clinical skills learning centers), universities and medical schools (including the clinical skills learning center and classrooms), and health centers arena/ comprehensive health services (including outpatient education and slightly community-based education) were identified for clinical education. In addition, in the second map, the worlds and social discourses residing in these arenas include (clinical teachers and educators, students, hospital physicians, residents, nurses, non-educational staff, family physicians, and simulation and technology) were represented. According to the third map (positional map), the challenges and problems related to clinical training in undergraduate medical education in Iran were illustrated in six positions, which include challenges and problems of curriculum (position 1), challenges and problems related to culture, behavior and attitude in clinical education (position 2), challenges and problems of management and

leadership in clinical education (position 3), challenges and problems related to the environment, space and time in clinical education (position 4), challenges and problems of financial in clinical education (position 5) and challenges and problems related to equipment and technology in clinical education (position 6). Finally,, elements and recommendations were provided based on this map to develop and support effective clinical teaching [27].

Findings of third sub-study

Expert panel findings

Ten experts (6 medical education specialists and four experienced clinical teachers) were present in the expert panel. Descriptive findings of them is provided in Table (2).

Table 2. Number and distribution of expert panel according to gender, field of expertise and academic rank

Descriptive statistics		N	Percentage
**Panel members in terms of medical and clinical education**			
Medical education specialists		6	% 60
Experienced clinical teachers		4	% 40
**Gender**			
Medical education specialists	Male	3	% 50
	Female	3	% 50
Experienced clinical teachers	Male	3	% 75
	Female	1	% 25
**Specialized field and teaching practice**			
Medical education specialists	Clinical education	2	33/3
	Curriculum planning and program evaluation	1	16/7
	Teaching-learning theories	2	33/3
	Student assessment	1	16/7
Experienced clinical teachers	Gastroenterology	1	% 25
	Social medicine	1	% 25
	Pediatrics	1	% 25
	Surgery	1	% 25
**Academic rank**			
Medical education specialists	Assistant Professor	3	% 50
	Associate Professor	3	% 50
	Full Professor	-	-
Experienced clinical teachers	Assistant Professor	1	% 25
	Associate Professor	2	% 50
	Full Professor	1	% 25

In the first step of the third sub-study, based on the expert panel method, the results of the first and second sub-studies were combined with a deductive approach. In this sub-study, the model

obtained from the first sub-study was strengthened and developed based on the elements that emerged in the second sub-study, and an effective clinical teaching-learning model was developed for undergraduate medical education in Iran (see Figure 2).

Figure 2. The final model of effective clinical teaching-learning for undergraduate medical education based on the synthesis of the results of the first and second sub-studies in the expert panel

Based on the expert panel discussions, individuals such as residents, hospital physicians (specialist and attending physicians), nurses, and non-educational staff present in the context of clinical education were considered and included in the final model. According to Clark's methodology, these individuals are on the margin of teaching-learning situations, meaning they do not formally educate students but are involved in teaching-learning in opportunistic situations. Therefore, they assist students in achieving learning goals and outcomes through hidden and informal teaching.

In addition, based on the synthesis of the results of the first and second sub-studies in the expert panel discussion, the main arenas of teaching-learning in the clinical training in undergraduate medical education were considered in the final model. These arenas include hospitals, medical schools and health centers (comprehensive health services). Accordingly, in the arenas of clinical teaching-learning, or more generally in the context of clinical education, different social worlds influence the teaching-learning discourse.

Also, based on the combination of the results of the first and second sub-studies in the expert panel discussion, the component of teaching-learning culture is formed from the intersection of two behavioral dimensions (student, teacher and patient) and social (interaction between student-instructor-patient). Teaching-learning culture is at the heart of the clinical context and as part of this system (clinical environment culture) impacts the students' achievement or non-achievement of clinical learning outcomes. Clinical teaching-learning culture can be influenced

by residents, hospital physicians (specialist and attending physicians), nurses, non-educational staff, as well as the social worlds of these people.

Moreover, based on the synthesis of the first and second sub-studies in the panel discussion, the financial dimension was added to the final model as a factor that affects effective teaching-learning. Lack of funding for education is one of the obstacles to effective teaching-learning in clinical training in undergraduate medical education in Iran. In other words, by providing financial resources, the quality and effectiveness of clinical education can be increased.

Based on the model's modification and development in the expert panel's discussion, the technology dimension with three subcategories (distance clinical education, simulation and technologies-enhanced learning) was added to the final model.

Unlike the results of the synthesis of qualitative studies (the first sub-study), which identified and categorized teachers' knowledge and skill in information and communications technology as one of the components of an effective teaching-learning framework, this sub-study, based on expert panel discussion, presented a more developed perspective of technology in clinical education and technology in clinical education was considered as one of the components of an effective teaching-learning framework. This importance and emphasis was the effect of COVID-19 on medical education.

In fact, most of the discussion in the expert panel was about clinical education during the COVID-19 era and after the post-pandemic. This critique noted the model obtained from the first sub-study, which did not consider the technology dimension alongside other dimensions. This critique noted the model obtained from the second sub-study, which did not consider the technology dimension alongside other dimensions. Finally, the PCC-Best conceptual model was developed, which includes seven dimensions of pedagogical, context, content, budget, educational leadership, social, and technological, and can be used to conceptualize, design, and organize clinical teaching-learning in undergraduate medical education in Iran. The naming of

the conceptual model obtained from this research is based on the acronym of seven dimensions, which is presented below.

P (Pedagogical) **C** (Context) **C** (Content) - **B** (Budget) **e** (Educational leadership) **s** (Social) **t** (Technology)

In the next step, the framework synthesis was performed based on the best fit, and an operational framework was conceptualized for the proposed model in relation to effective teaching-learning in clinical education. The results are presented below.

Framework synthesis based on the best fit

The framework synthesis based on best fit requires identifying a framework relevant to the research subject. The framework of Ottenhoff- de Jonge et al. is one of the frameworks that conceptualized teaching and learning in medical education [12]. Because this framework best fits the current study, it served as the foundation for data analysis in this section of the study. The analysis of the experts' answers to the eight open-ended questions about teaching and learning was relevant and in line with the original framework. We preserved the option of changing the dimensions of the primary framework open based on prospective expert opinions. The experts' responses, which were short recorded audios, were first transcribed on paper, then read and re-read to identify the areas of meaning that reflected participants' orientation and beliefs. In this way, their understanding and conceptualization in relation to teaching and learning and the meaning of the dimensions of the research model were labeled according to the dimensions of the framework of Ottenhoff- de Jonge et al., [12]. The fragments extracted from texts which did not cover exclusively one of the dimensions of the original framework or were the common point of both dimensions were added to the primary framework as a new dimension. In contrast to Ottenhoff-de Jonge et al., [12], the new framework incorporates a three-dimensional epistemological orientation matrix on which the seven dimensions of the final model obtained by the expert panel are presented (see Table 3).

Table 3. Dimensions of effective teaching-learning in clinical education based on the range of epistemological orientations

N	Dimensions	Range of epistemological orientations		
		**Teaching-centered orientation**	**Learning-centered orientation**	**Teaching-learning centered orientation**
1	**Pedagogical**<br>- Curriculum planning, objectives and desired learning outcomes, teaching approaches and methods, assessment and learning opportunities	Teacher-centered	Student-centered	- Teacher facilitation<br>- Student centered<br>- Critical pedagogy (patient involvement in clinical teaching)
2	**Content or behavioral**<br>- Positive personality traits of students (motivation, communication skills, love and interest to learning, etc.)<br>- Development of student' autonomy and self-direction<br>- Preparation, knowledge, skills and previous experiences of the student<br>- Positive personality traits of the teacher (interest and enthusiasm, motivation, humor, etc.)<br>- Teacher experience, knowledge and clinical ability<br>- Professionalism and role modeling of clinical teacher<br>- Individual characteristics of patients<br>- Patient problems and their educational value	Teacher' action, work and behavior in the center	Student' action, work and behavior in the center	Teacher, student, patient and triangular behavioral system
3	**Social**<br>- Teacher-student-patient interaction<br>- Multidisciplinary team-interactive approach in clinical teaching and care<br>- Collaborative teaching-learning<br>- Worlds and social discourses present in the arenas (students, teachers, patients, residents, specialist and attending physicians, nurses and non-educational staff)	One-sided by the teacher	Mutual to maintain student' attention and focus	Network and interactional to negotiate and build a collaborative community
4	**Technology (clinical teaching based on new technologies)**	for transmitting unstructured information and knowledge	Lack of educational design, for opportunistic learning	Integration in face-to-face clinical training to achieve learning outcomes
5	**Budget and financial**	Serves to external motivation of the teacher	Not emphasized (not important)	Equipping and strengthening teaching-learning settings in clinical education
6	**Context**<br>- Creation and promoting of a positive, conducive and non-threatening learning environment<br>- Strong teaching-learning culture<br>- Arenas and worlds and discourses present in them	Not emphasized (not important)	In order to promoting relaxation in the students is somewhat emphasized	Highly important / focus on context as a full- range and comprehensive educational architecture
7	**Educational leadership**	Autocratic, authoritarian and tyrannical	Delegative or laissez-faire leadership	Democratic and participative leadership

Although some components of our framework are common to the framework of Ottenhoff- de Jonge et al., [12], our framework and research data provide a more extended and holistic perspective. Therefore, the new framework (Table 3) consists of three epistemological orientations of teaching and learning. These epistemological orientations include Teaching-centered, learning-centered and teaching-learning centered, which are defined and arranged in a column. This matrix shows the seven dimensions of effective teaching-learning in clinical education in a row. Pedagogical, content or behavioral, social, technological (clinical teaching using new technologies), budget and financial, context, and educational leadership are all included.

As seen in the proposed matrix, each of the seven dimensions has its own meaning in terms of epistemological orientation. For example, the pedagogical dimension in the teaching-centered epistemological orientation is teacher-centered. Meanwhile, it is student-centered in the learning-centered orientation, and the pedagogical dimension in the teaching-learning-centered orientation means teacher facilitation and student-centered. Additionally, critical pedagogy (patient involvement in clinical teaching) is also taken into account. Thus, in this matrix, the meaning of other dimensions is presented in the three epistemological orientations: teaching-centered, learning-centered, and teaching-learning centered. Furthermore, based on this matrix, effective teaching-learning and achieving learning objectives could not be implemented in terms of the significance of each of these dimensions in both teaching-centered orientation and learning-centered orientation. For example, in teaching-centered orientation, the social dimension is a one-way flow by the teacher, which will not lead to the creation of an effective teaching-learning process. At the same time, in the learning-centered orientation, the application of technology in clinical teaching without educational design can only serve opportunistic learning. Accordingly, in Figure (3), the operational framework for effective teaching-learning entitled (ECT-TLCO) based on the best fit approach was conceptualized.

E (Effective) **C** (Clinical) **T** (Teaching)- **T** (Teaching) **L** (Learning) **C** (Centered) **O** (Orientation)

Figure 3. Conceptualizing the operational framework of effective teaching-learning based on the best fit approach

In fact, the meaning of this framework is that effective clinical teaching can be implemented in terms of the epistemological orientation of the teaching-learning centered.

Finally, while Ottenhoff-de Jonge et al's framework [12] served as the basis for the construction of our research framework, the two are similar in some ways and different in others. Table (4) compares the dimensions and components of these two frameworks.

Table 4. The comparison of the dimensions of the new framework and the framework of Ottenhoff- de Jonge et al., (2021)

Dimensions	Comparison of the new framework to the Ottenhoff- de Jonge et al framework
**Desired learning outcomes**	This dimension is identical in both frameworks but developed in the new framework as the pedagogical dimension (curriculum planning, objectives and desired learning outcomes, teaching approaches and methods, assessment and learning opportunities).
**Expected use of knowledge***	Not applicable for the new framework
**Responsibility for transforming knowledge***	Not applicable for the new framework
**Nature of knowledge***	Not applicable for the new framework
**Students' motivation** and **Students' previous and existing conceptions** and **Students' professional development**	To some extent, these two frameworks have a common language in these components. The previous framework focused on the students' motivation, their previous and existing conceptions and students' professional development, While the new framework considers the behavioral system of students, teachers and patients and has been extended in the content or behavioral dimension (such as students' motivation, preparation, knowledge, skills and previous experiences of the student, development of student' autonomy and self-direction, teacher' motivation, individual characteristics of patients, patient problems and their educational value and etc.)
**Teacher-student interaction**	This dimension is identical in both frameworks, but it has been extended in the new framework as the social dimension (including; teacher-student-patient interaction, worlds and social discourses present in the arenas and etc.)
	This dimension is the same in both frameworks, but it has been developed in the new framework as the context dimension

**Creation of a conducive learning environment**	(including; the creation and promotion of a positive, conducive, and non-threatening learning environment, teaching-learning solid culture and arenas and worlds and discourses present in them)
**Technology (clinical teaching based on new technologies)****	This dimension is not considered in the previous framework
**Budget and financial****	This dimension is not considered in the previous framework
**Educational leadership****	This dimension is not considered in the previous framework

* Not applicable for the new framework   ** New

As seen in Table (4), the dimensions and components of the two frameworks are compared. One of the most important differences between the framework of Ottenhoff- de Jonge et al., [12] and the new framework is that, unlike the previous framework, which suggested a two-dimensional matrix, the new framework provides a three-dimensional matrix for epistemological orientations in connection to teaching and learning.

Furthermore, some dimensions of the new framework, such as expected use of knowledge, responsibility for transforming knowledge, and nature of knowledge, are not applicable because Ottenhoff-de Jonge et al., [12], conceptualized medical educators' beliefs about teaching, learning, and knowledge in their study, whereas our study only focuses on teaching-learning. Finally, in the new framework, three dimensions of technology (clinical teaching based on new technologies), budget and financial and educational leadership have been considered, which have not been addressed in the previous framework.

Discussion

The first sub-study of this research synthesized elements and dimensions of effective teaching-learning in five dimensions based on a systematic review of qualitative studies and meta-synthesis. The second sub-study provided the teaching-learning situation in undergraduate medical education's clinical training, focusing on its challenges, utilizing Clarke's situational analysis approach. The conceptual model (PCC-Best) was developed in seven dimensions of pedagogical, context, content, budget, educational leadership, social, and technology in the third sub-study, based on the modification and development of the model in the expert panel,

and teaching-learning in the clinical training of undergraduate medical education can be conceptualized, designed, and organized based on it.

Based on research conducted for successful clinical education in the literature, dimensions and components such as the tutor and student's roles, the patient's roles and their characteristics, and the characteristics of a good clinical environment are considered [4]. Ross & Stenfors-Hayes [6] consider elements such as teacher and teaching activities, learner and learning activities, shared activities between teacher and learner, teaching and learning situations, and content for teaching activities in undergraduate medical education.

In our research, a conceptual model entitled (PCC-Best) was presented. Although this model includes elements and dimensions such as pedagogy, content, or behavioral (teacher, student, and patient and their characteristics), context, and environment, it is more complex and developed than previous research results and includes other elements and dimensions such as budget, educational leadership, social, and technology for the best and most effective clinical teaching.

Furthermore, the framework synthesis in this study was carried out using one of the available frameworks in the literature [12] that best fit the subject of our research. Finally, based on this approach (best fit), an operational framework entitled (ECT-TLCO) was conceptualized for effective teaching-learning.

Although the new framework of this study confirms some of the old framework's findings, dimensions, and components, it also contains significant differences. One of the important differences between this study and the study of Ottenhoff- de Jonge et al., [12] is related to the study context. They developed their framework, emphasizing the preclinical teaching context, whereas the current study focuses on the clinical context. Another difference between the new framework and the previous one is that the framework of this research offers a more extended perspective of the teaching-learning dimensions. In other words, in addition to conceptual

development, in the new framework, there are dimensions such as budget and financial, technology and educational leadership that has not been addressed in the previous framework. Another difference is that some components of the previous framework are not applicable to the new framework; this is because the old framework, in addition to teaching and learning, also considered knowledge belief orientation.

Another important difference between the new framework and the previous one is the shape and structure of the matrix. In the previous framework, the belief orientation was a two-dimensional matrix, while in the new one, the belief and epistemology orientation is three-dimensional. In fact, the previous framework took into account teaching-centered and learning-centered orientations, whereas the new framework also takes into account teaching-learning centered orientation. We believe that effective teaching-learning may be implemented using this epistemological perspective (teaching-learning oriented) because teaching and learning are two sides of the same coin. In this regard, Thomas Angelo says, "teaching in the absence of learning and without learning is a futile activity." Therefore, the effectiveness of teaching reflects the learning rate of students [28]. In general, with the epistemological orientation of teaching-learning-centered and extensions of seven dimensions in this orientation (such as teacher facilitation, student-centered and critical pedagogy/ patient involvement in clinical teaching, focus on the teacher, student, patient behavioral system, networked social interactions and build a collaborative community, integration of technology in face-to-face clinical training, equipping and strengthening teaching-learning settings, focus on context as a full-scale educational architecture, and democratic and participative leadership), effective teaching-learning can be implemented in clinical education.

In terms of the clinical environment and the extensions of the seven dimensions in the teaching-learning focused orientation, it can be argued that the teaching-learning triangle is formed by the teacher, student, and patient. To create effective teaching-learning, it is essential to focus

on these three. Educational policymakers should make this possible by selecting motivated and interested students in the medical profession. Throughout the course, students' self-directed learning skills must be strengthened and their development as lifelong learners. Students' learning in clinical settings and contexts is highly dependent on emotional, educational, and organizational support [29-31], which should not be overlooked.

The teacher is an important part of the educational program [32-35]. Accordingly, teachers and educators have an important role in students' clinical learning. Thus, recruiting competent teachers is significant in medical education, and their personal and professional development must be taken into account during the service.

Contacting real patients plays an essential role in educating students, teachers, and physicians [36-38]. Patients should not be seen as merely “subjects for teaching-learning” Educational policymakers and clinical educators should involve patients in clinical education, curriculum design, or evaluation. In other words, a culture of patient involvement in education must be established, and patients' voices must be heard in the educational process.

Generally, patients prefer participating and being involved in the clinical teaching process. Basically, teaching with patients allows three key domains of learning to be integrated with teaching [39]: A) clinical (knowledge and skills); B) Professional character or professionalism (teamwork and ethical considerations); C) Communications (with staff and patients).

Interaction with some patients is difficult for medical students, especially if the patient is hostile, angry, uncooperative, disinterested, overly talkative, or has chronic pain [40].

Interaction in clinical learning environments is crucial. Interactions in the clinical environment should be considered "Key teaching moments" along with opportunities for tutors to help students develop competence in communication skills [41]. In clinical education environments and settings, the principles of constructivist theories and adult learning can be the basis for teaching-learning. Accordingly, the use of collaborative learning strategies such as small group

teaching, problem-based learning, team-based learning, peer learning, etc. can be great mechanisms. In fact, it is only through participation that new methods are learned and new tasks are gradually performed [42]. In addition, in these environments, conditions must be provided for students to build their own knowledge as adult learners.

Contrary to popular belief, leaving learners and students in a clinical setting has no pedagogical basis. It is better for clinical teachers to be equipped with pedagogical knowledge. Pedagogical knowledge is a term used for knowledge of how to teach that can be used in a wide range of educational fields. Therefore, in clinical teaching, educational design and even planning of teaching-learning opportunities are of great importance [43]. In this regard, engaging in faculty development programs effectively develops them.

Regarding the technology dimension, it can be said that although simulations and new technologies such as virtual reality, augmented reality, virtual patient, etc., are increasingly used in health professions education, the long-held tradition of teaching with the engagement of real patients remains valuable [42]. Modern technologies in medical education are important because they have been able to guide opportunistic and informal learning in clinical settings and create a constructive alignment between this type of learning and formal educational activities [44]. Accordingly, the integration of technology in face-to-face clinical education is important.

The context, in general, and the educational environment or atmosphere, in particular, are the other dimensions. Various educational theorists have emphasized the importance of context in education. Michael Allen [45] emphasizes the importance of context in the design of learning interventions. He notes, "in many ways, context is both the most fundamental component of education and often frequently neglected."Colvin Clark [46] refers to the focus on the context in education as an immersive architecture or whole-task instructional design. In general, context is important in medical education, and it can be said that when information is applied

to a situation by a person, a dynamic interaction occurs. Ignoring the environment and situation in which knowledge is applied is metaphorically similar to "focusing (only) on the hammer" when nailing to a wall or board. In this state, the nail used, the wall or board to which the nail is affixed, is neglected. The learning environment or "educational atmosphere" as one of the components of context is one of the key aspects of the curriculum that is less tangible than other aspects of the curriculum. According to Genn [47], the "educational climate" is the soul of the medical curriculum. In this regard, clinical teachers, educators, and curriculum planners should consider measuring the educational environment as part of curriculum evaluation and promote an appropriate learning-learning environment. Training within the clinical settings, such as bedside teaching, inpatient education, outpatient clinic, and community education, is at the heart of healthcare education and provides a vital component of clinical education. This training guides students in the clinical environment's culture and social aspects of the clinical environment and shapes students' professional values to prepare them for future work and activity [48, 42]. Medical teachers and educators are engaged in a wide range of activities, including teaching-learning, curriculum development, assessment and evaluation, and team and program management. All of these activities need leadership in some way [49]. Therefore, they must be prepared for this important role. In addition, teaching-learning in clinical settings requires participatory leadership. Participation in leadership and management training courses is encouraged in this regard in order to develop them.

Finally, financial support is particularly important in clinical education [50]. Resources to purchase educational materials and technology and not equipping teaching-learning environments in clinical education are important financial constraints and can impair the quality and effectiveness of teaching-learning.

Limitations

While there may be valid articles, studies, and frameworks in the literature (other than Persian and English) that were not included in this research, searching for articles and studies in the first sub-study and finding the relevant framework and model in the literature to framework synthesis in the third sub-study was based on Persian and English (one of the inclusion criteria). Other limitations of this study were related to semi-structured telephone interviews in the second sub-study. Two participants did not allow their voices to be recorded, and the interviewer was forced to write down the conversation in these two interviews during the interview. Another limitation of this research is related to the context in the third sub-study. The specific cultural differences and characteristics may have influenced epistemological beliefs about teaching-learning in clinical education. For this reason, caution should be taken in transferring this epistemological and classified framework to the context of other countries. Finally, some experts did not respond to the question-based phase questions submitted to them via the Porsline (web-based) due to their busy schedules. As a result of this lack of cooperation, they also did not participate in the rounds of the expert panel.

Conclusions

While presenting the dimensions of effective teaching-learning in clinical education based on a multi-method study, a new framework in relation to epistemological orientations about teaching and learning was developed in this study, based on which each of these dimensions can be conceptualized, and finally, an effective teaching-learning system in clinical medicine education was implemented. In this study, researchers presented a new framework for epistemological beliefs about teaching and learning, based on the framework of Ottenhoff- de Jonge et al. [12] on medical educators' beliefs about teaching, learning, and knowledge. The new framework is a three-dimensional matrix based on which the dimensions of effective teaching-learning in clinical education were explained. Each dimension has a special meaning

in terms of epistemological orientation about teaching-learning. Implementing effective teaching-learning in clinical medical education requires moving from the single teaching-centered or learning-centered orientation to the teaching-learning centered orientation. Focusing on the seven dimensions based on the epistemological orientation of teaching-learning is the starting point of effectiveness and improving the quality of clinical education. In order to implement the model developed through this research and the teaching-learning orientation, all the following items should be given serious consideration: selecting motivated students and strengthening their motivation during education; comprehensive development of students during education; training students as lifelong learners; recruitment, employment and retention of competent teachers and their personal and professional development; implementing an educational culture of involving patients in clinical education; use of collaborative teaching-learning strategies; equipping clinical teachers with pedagogical knowledge and motivating clinical teachers and educators to engaging in faculty development courses and programs in medical education, integration of new technologies in medical education (special attention to technology-enhanced clinical education), focus on context and environment and promoting the positive teaching-learning environment, developing educational leadership and management skills of clinical teachers and educators, participative leadership in the clinical environment and funding of medical education.

Acknowledgements

The research team are grateful the clinical teachers, medical education specialists, and medical students who participated in this study. Also, we thank and appreciate Dr. Mohammad Palesh, Dr. Mehrnoosh Khoshnoodifar (internal reviewers), Dr. Mitra Amini and Dr. Mohammad-Ali Hosseini (external reviewers); and Dr. Masomeh Kalantarion and Dr. Nasim Gheshlaghi (peer students) for their valuable and useful comments and feedback.

Author Contributions

Conceptualization: Hamed Khani, Soleiman Ahmady, Shirdel Zandi.

Data curation: Hamed Khani, Shirdel Zandi, Mojgan Mohammadimehr.

Formal analysis: Hamed Khani, Shirdel Zandi, Babak Sabet, Mojgan Mohammadimehr.

Funding acquisition: Not applicable

Investigation: Hamed Khani, Soleiman Ahmady.

Methodology: Hamed Khani, Soleiman Ahmady, Shirdel Zandi, Babak Sabet, Mojgan Mohammadimehr, Somayeh Niakan.

Project administration: Soleiman Ahmady.

Resources: Hamed Khani, Shirdel Zandi.

Software: Hamed Khani, Mojgan Mohammadimehr.

Supervision: Soleiman Ahmady, Babak Sabet.

Validation: Hamed Khani, Mojgan Mohammadimehr, Somayeh Niakan.

Visualization: Hamed Khani, Shirdel Zandi.

Writing – original draft: Hamed Khani, Shirdel Zandi.

Writing – review & editing: Hamed Khani, Soleiman Ahmady, Babak Sabet, Mojgan Mohammadimeh, Somayeh Niakan.

Funding statement

The authors received no specific funding for this work.

Data and materials availability statement

The data that support the findings of this study are not publicly available, but can be available from the authors on reasonable request. All datasets generated and analyzed and other materials during the current study (such as; qualitative interviews questions; expert panel questions/question-centered and discussion phase; and open-ended questions related to

epistemological orientation towards teaching and learning) are available from the corresponding author on reasonable request.

Endnotes

[1]. Preferred Reporting Items for Systematic Reviews and Meta-analyses

[2]. Population, Intervention, Comparison, Outcome, and Study design

References

1. Ramani S, Leinster S. AMEE Guide no. 34: Teaching in the clinical environment. Med Teach. 2008; 30(4):347-64. https://doi.org/10.1080/01421590802061613
2. Spencer J. Learning and teaching in the clinical environment. BMJ. 2003;326:591-4. https://doi.org/10.1136/bmj.326.7389.591
3. Torkzadeh J, Mohammadi M, Sutoodehfar S. Developing and Validating of the Teaching-Learning Comprehensive System in Higher Education. J Curric Stud. 2015;4(2):93-116. https://doi.org/10.22099/jcr.2015.3088
4. El Bingawi HM, Alghamdi AH. Clinical Teaching: Sixty Tips for Successful Implementation. Sudan j med sci. 2020;15(3):313-23. https://doi.org/10.18502/sjms.v15i3.6964
5. Ernstzen DV, Bitzer E, Grimmer-Somers K. Physiotherapy students' and clinical teachers' perceptions of clinical learning opportunities: A case study. Med Teach. 2009;31(3):e102-15. https://doi.org/10.1080/01421590802512870
6. Ross MT, Stenfors- Hayes T. Development of a framework of medical undergraduate teaching activities. Med Educ. 2008;42(9):915-22. https://doi.org/10.1111/j.1365-2923.2008.03147.x
7. Kember D. A reconceptualisation of the research into university academics' conceptions of teaching. Learn Instr. 1997;7(3):255-75. https://doi.org/10.1016/S0959-4752(96)00028-X
8. Postareff L, Lindblom-Ylänne S. Variation in teachers' descriptions of teaching: Broadening the understanding of teaching in higher education. Learn Instr. 2008;18(2):109-20. https://doi.org/10.1016/j.learninstruc.2007.01.008

9. Samuelowicz K, Bain JD. Conceptions of teaching held by academic teachers. High educ. 1992;24(1):93-111. https://doi.org/10.1007/BF00138620

10. Samuelowicz K, Bain JD. Revisiting academics' beliefs about teaching and learning. High educ. 2001;41(3):299-325. https://doi.org/10.1023/A:1004130031247

11. Wu, Pi-Ju. A Qualitative Evidence Synthesis of Studies on Teaching: Focusing on Student Learning, Conference ECER. 2019. https://eera-ecer.de/ecer-programmes/conference/24/contribution/47426/

12. Ottenhoff-de Jonge MW, van der Hoeven I, Gesundheit N, van der Rijst RM, Kramer AW. Medical educators' beliefs about teaching, learning, and knowledge: development of a new framework. BMC Med Educ. 2021;21(176):1-13. https://doi.org/10.1186/s12909-021-02587-x

13. Sandelowski M, Barroso J. Handbook for synthesizing qualitative research. Springer publishing company; 2006;24. https://parsmodir.com/wp-content/uploads/2020/03/MetaSynBook.pdf

14. Maxwell JA. Designing a qualitative study. The SAGE handbook of applied social research methods. 2008;2:214-53.

15. Maxwell JA. Qualitative research design: An interactive approach. Sage publications; 2012;8.

16. Kvale S. Validation as Communication and Action: On the Social Construction of Validity. Paper presented at the Annual Meeting of the American Educational Research Association. NewOrleans, LA,1994.

17. Clarke AE. Situational Analysis: Grounded Theory after the Postmodern Turn. Thousand Oaks: Sage; 2005.

18. Lincoln YS, Guba EG. Naturalistic inquiry. Sage; 1985.

19. Galliers RD, Huang JC. The teaching of qualitative research methods in information systems: an explorative study utilizing learning theory. Eur J Inf Syst. 2012;21(2):119-34. https://doi.org/10.1057/ejis.2011.44

20. Fitch K, Bernstein SJ, Aguilar MD, Burnand B, LaCalle JR, Lazaro P, et al. The RAND/UCLA appropriateness method user's manual. Rand Corp Santa Monica CA; 2001;1. https://www.rand.org/pubs/monograph_reports/MR1269.html

21. Barnett-Page E, Thomas J. Methods for the synthesis of qualitative research: a critical review. BMC Med Res Methodol. 2009;9(59):1-11. https://doi.org/10.1186/1471-2288-9-59

22. Kelly M, Morgan A, Ellis S, Younger T, Huntley J, Swann C. Evidence based public health: A review of the experience of the National Institute of Health and Clinical Excellence (NICE) of developing public health guidance in England. Soc Sci Med. 2010;71(6):1056-62. https://doi.org/10.1016/j.socscimed.2010.06.032

23. Carroll C, Booth A, Leaviss J, Rick J. "Best fit" framework synthesis: refining the method. BMC Med Res Methodol. 2013;13(37):1-16. https://doi.org/10.1186/1471-2288-13-37

24. Kember D, Gow L. Orientations to teaching and their effect on the quality of student learning. J Higher Educ. 1994;65(1):58-74. https://doi.org/10.2307/2943877

25. Trigwell K, Prosser M. Changing approaches to teaching: A relational perspective. Stud High Educ. 1996;21(3):275-84. https://doi.org/10.1080/03075079612331381211

26. Ahmady S, Khani H. The Development of the Framework of Effective Teaching-Learning in Clinical Education: A Meta-Synthesis Approach. Educ Res Int. 2022:28;2022:1-12. https://doi.org/10.1155/2022/4751931

27. Ahmady, S., Khani, H. The situational analysis of teaching-learning in clinical education in Iran: a postmodern grounded theory study. BMC Med Educ. 2022:22(1),520: 1-15. https://doi.org/10.1186/s12909-022-03577-3

28. Sajjad S. Effective teaching methods at higher education level. Pak J Spec Educ. 2010;11:29-43.

29. Morris C, Cook V, Daly C, Newman M. Replacing 'the firm': re-imagining clinical placements as time spent in communities of Practice. Work based learning in clinical settings--insights from socio-cultural perspectives. Oxford: Radcliffe Medical. 2012.

30. Blakey AJ. Cultivating student thinking and values in medical education: What teachers do, how they do it and who they are?. Doctoral dissertation, University of Otago. 2016.

31. Dornan T, Tan N, Boshuizen H, Gick R, Isba R, Mann K, Scherpbier A, Spencer J, Timmins E. How and what do medical students learn in clerkships? Experience based learning (ExBL). Adv Health Sci Educ. 2014;19(5):721-49. https://doi.org/10.1007/s10459-014-9501-0

32. Raingruber B, Bowles K. Developing student evaluation instruments to measure instructor effectiveness. Nurse Educ. 2000;25(2):65-9. https://doi.org/10.1097/00006223-200003000-00007

33. Salsali M. Evaluating teaching effectiveness in nursing education: An Iranian perspective. BMC Med Educ. 2005;5(29):1-9. https://doi.org/10.1186/1472-6920-5-29

34. Okoronkwo IL, Onyia-Pat JL, Agbo MA, Okpala PU, Ndu AC. Students' perception of effective clinical teaching and teacher behaviour. Open J Nurs. 2013;3:63-70. https://doi.org/10.4236/ojn.2013.31008

35. D'Costa MP, Swarnadas GS. Students' perceptions of effective clinical teaching skills and teacher behaviours on learning. Manipal Journal of Nursing and Health Sciences (MJNHS). 2016;2(2):1-8.

36. Wykurz G, Kelly D. Developing the role of patients as teachers: literature review. BMJ. 2002;325:818-21. https://doi.org/10.1136/bmj.325.7368.818

37. Towle A, Godolphin W. A meeting of experts: the emerging roles of non-professionals in the education of health professionals. Teach High Educ. 2011; 16(5):495-504. https://doi.org/10.1080/13562517.2011.570442

38. Towle A, Farrell C, Gaines ME, Godolphin W, John G, Kline C, et al. The patient's voice in health and social care professional education: the Vancouver statement. Int J Health Gov. 2016;21(1):18–25. https://doi.org/10.1108/IJHG-01-2016-0003

39. Janicik RW, Fletcher KE. Teaching at the bedside: a new model. Med Teach. 2003;25(2):127-30. https://doi.org/10.1080/0142159031000092490

40. Shapiro J, Rakhra P, Wong A. The stories they tell: How third year medical students portray patients, family members, physicians, and themselves in difficult encounters. Med Teach. 2016;38(10):1033-40. https://doi.org/10.3109/0142159X.2016.1147535

41. Steinauer JE, O'Sullivan P, Preskill F, Ten Cate O, Teherani A. What makes "difficult patients" difficult for medical students?. Acad Med. 2018;93(9):1359-66. https://doi.org/10.1097/ACM.0000000000002269

42. Burgess A, van Diggele C, Roberts C, Mellis C. Key tips for teaching in the clinical setting. BMC Med Educ. 2020;20(2):1-7. https://doi.org/10.1186/s12909-020-02283-2

43. Steinert Y, Basi M, Nugus P. How physicians teach in the clinical setting: The embedded roles of teaching and clinical care. Med Teach. 2017;39(12):1238-44. https://doi.org/10.1080/0142159X.2017.1360473

44. Conn JJ, Lake FR, McColl GJ, Bilszta JL, Woodward- Kron R. Clinical teaching and learning: from theory and research to application. Med J Aust. 2012;196(8):527. https://doi.org/10.5694/mja10.11473

45. Allen MW, Sites R. Leaving ADDIE for SAM: An agile model for developing the best learning experiences. American Society for Training and Development. ASTD Press. 2012.

46. Clark RC. Evidence-based training methods: A guide for training professionals. American Society for Training and Development; ASTD Press. 2019.

47. Glenn JM. AMEE Medical education guide No 23. (Part 1) Curriculum, environment, climate, quality and change in medical education–a unifying perspective. Med Teach. 2001;23:337-44. https://doi.org/10.1080/01421590120063330

48. Sinclair S. Making doctors: an institutional apprenticeship. Routledge; 2020.

49. Dent J, Harden R, Hunt D. A Practical Guide for Medical Teachers, E-Book. Elsevier health sciences; 2021.

50. Bridges M, Diamond DL. The financial impact of teaching surgical residents in the operating room. Am J Surg. 1999;177(1):28-32. https://doi.org/10.1016/s0002-9610(98)00289-x

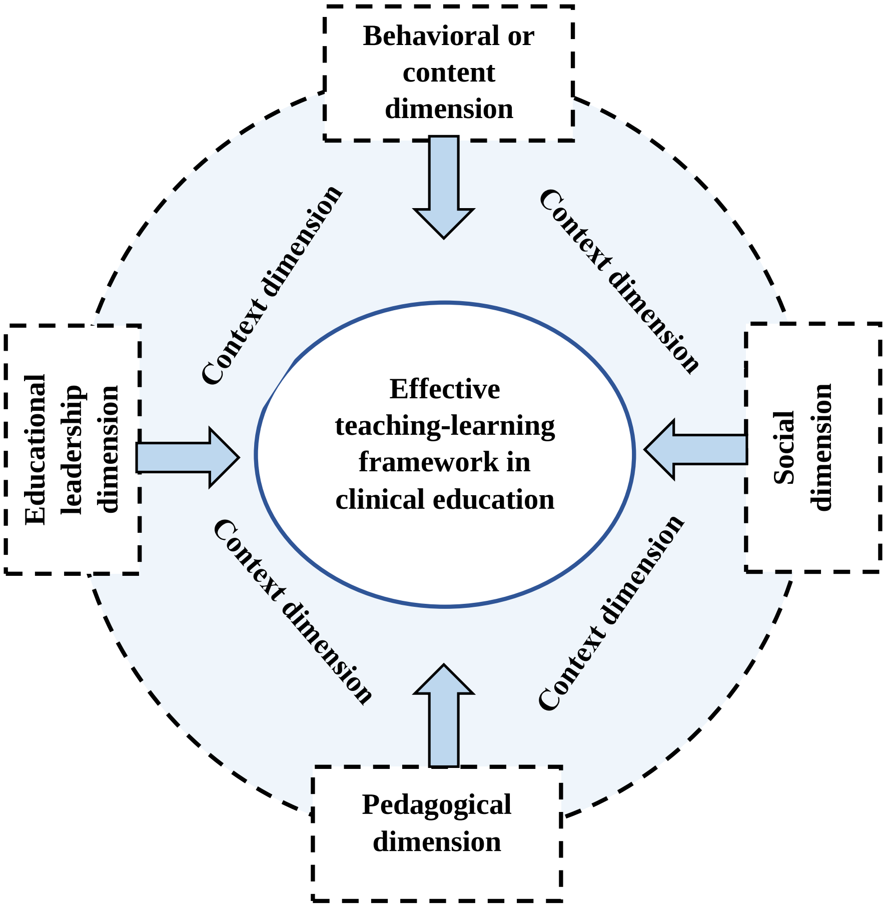

Behavioral or content dimension
Context dimension
Context dimension
Educational leadership dimension
Effective teaching-learning framework in clinical education
Social dimension
Context dimension
Context dimension
Pedagogical dimension

Figure 2

Click here to access/download;Figure;Figure 2.docx

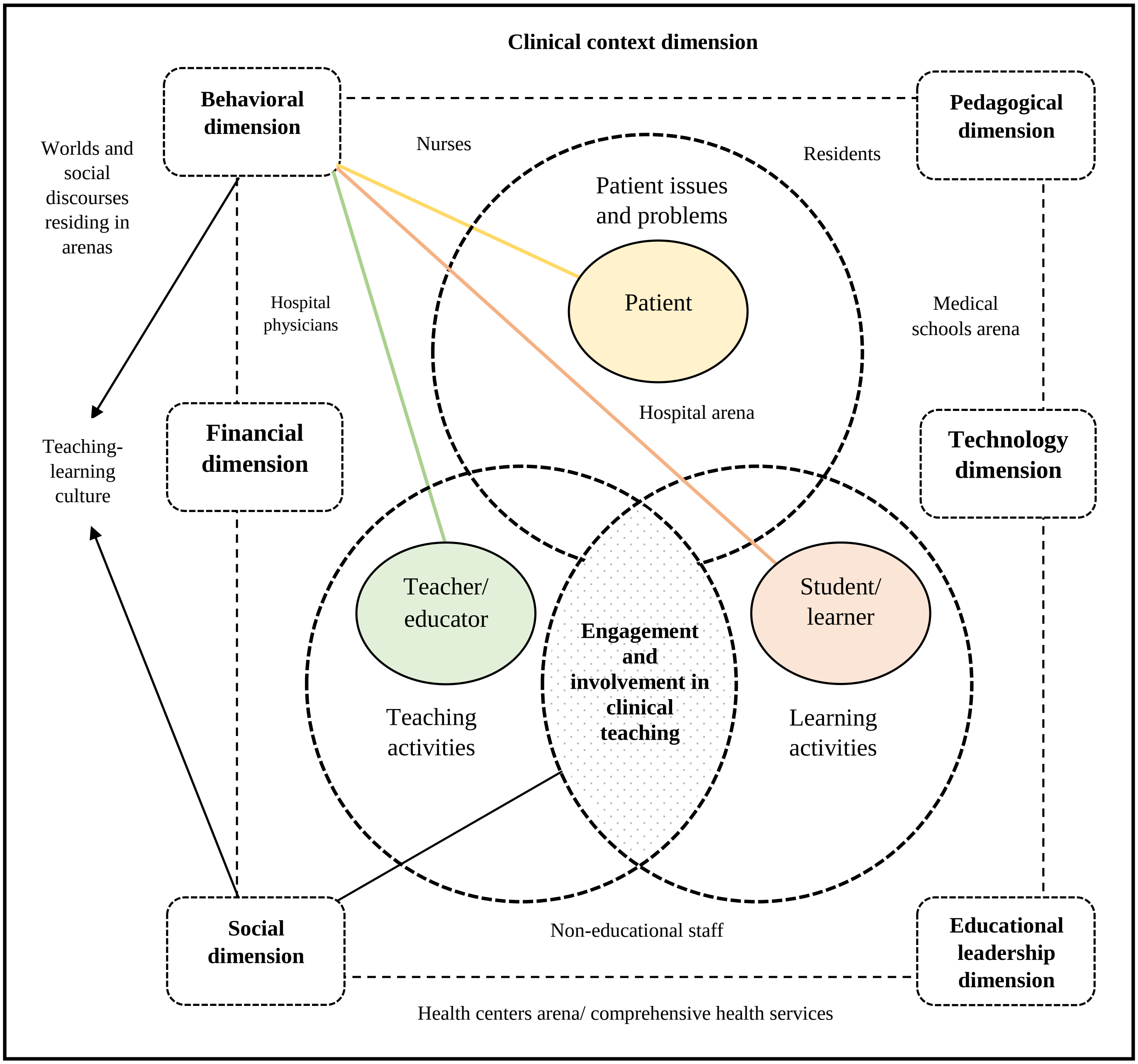

Click here to access/download;Figure;Figure 3.docx

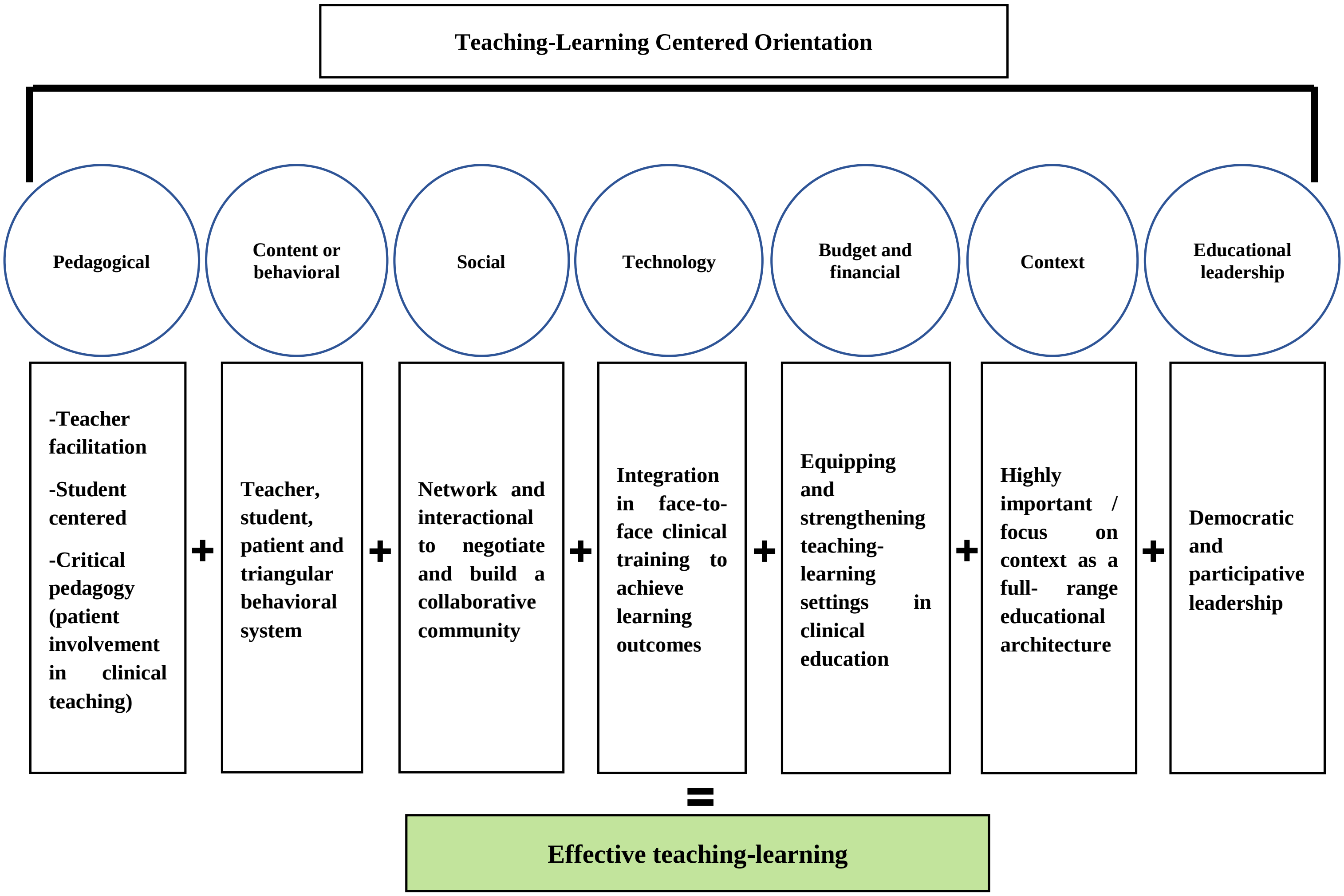

Supplement: Attachment — Submitted filename: PONE-D-23-10842.pdf [file pone.0289150.s004.pdf]
