# Supplementary material for: Teaching-learning in clinical education based on epistemological orientations: A multi-method study
Source: PLoS One. 2023 Nov 30;18(11):e0289150. doi: 10.1371/journal.pone.0289150 (PMC10688630; doi:10.1371/journal.pone.0289150)
Supplement: Attachment — Submitted filename: point-by-point response to reviewers.docx [file pone.0289150.s005.docx]

**Point-by-point response to reviewers**

**Manuscript Number:** PONE-D-23-10842R1

**Manuscript title: Teaching-learning in Clinical Education Based on Epistemological Orientations: A Multi-method Study**

**Dear Editor**

Thank you for giving us the opportunity to submit a revised draft of the manuscript "Teaching-learning in Clinical Education Based on Epistemological Orientations: A Multi-method Study" for publication in **PLOS ONE journal**.

We appreciate the time and effort that you and the reviewers dedicated to providing feedback on our manuscript and are grateful for the insightful comments on and valuable improvements to our paper.

We have carefully reviewed the comments and have revised the manuscript accordingly. Our responses are given in a point-by-point manner below. The changes of the paper have been uploaded in two versions including marked-up (revised manuscript with track changes) and unmarked (revised manuscript without tracked changes).

We hope the revised version is now suitable for publication and look forward to hearing from you in due course.

**Kind regards,**

**Corresponding Author**

**Reviewers' Comments to the Authors**

**Reviewer 1**

**Reviewer's comment:** Having read through your manuscript, I have identified various issues that do not align with the criteria set out for publication in this journal (As listed below). Also, they have been highlighted in the margin of the manuscript. Please address them point-by-point.

- The authors are supposed to expand this part to include a comprehensive review of the literature along with the previous studies. More importantly, presenting a theoretical framework is essential for the study.
- In a separate sub-heading the participants should be detailed. This allows the replication of the study by other researchers.
- The authors could put following sentences under a sub-heading, named design of the study. Also, they could explain why they chose this design for the readers.

(This study comprised three sub-studies conducted using a multi-method approach (Data and methods triangulation). This research data were collected through multiple methods such as synthesis of qualitative studies, literature review, analysis of the general medicine curriculum and related documents, remote semi-structured interviews (web-based and telephone), expert panel, and the framework synthesis based on the best fit. In addition to documents and curriculum analysis, experienced clinical teachers, medical education specialists, and eligible medical students participated in this study.)

- The seven-step method of Sandelowski & Barroso [13]…… These seven steps should be specified for readers.
- Please explain PICOS strategy for the readers.
- By two authors….. Who are they?
- The following criteria need clarification.

To ensure the data's trustworthiness, Maxwell [14, 15] criteria, such as descriptive, interpretative, and theoretical validity, as well as Kvale [16] criteria, such as pragmatic validity, were considered in this sub-study

- And five main open-ended questions…… What were those questions? How were they were designed and worded?
- All methods were conducted in accordance with the ethical principles of the Declaration of Helsinki……………………Please explain briefly ethical principles of Helsinki
- The discussion part needs to be restructured such that more justifications and explanations for attained findings be provided for the readers
- Please delete this part (other than Persian and English)

**Authors’ response:** We appreciate the time and effort that you dedicated to providing feedback on our manuscript and are grateful for your insightful comments. We have addressed your concerns as suggested by you point-by-point.

**The revised text reads as follows:**

**[**Teachers' belief orientation about teaching and learning should be considered to develop the framework of effective educational behaviors. "Beliefs" refer to perceptions and conceptions about teaching and learning, which are formed throughout life, deeply rooted, and contain cognitive and affective aspects (7, 8). The body of research shows that epistemological beliefs are one of the driving forces behind educators' educational behaviors and their choices in teaching design and delivery. In other words, how teachers teach and even teaching practice is unconsciously influenced by their conceptions and perceptions of teaching and learning (9, 10, 11, 12, 13, 14, 15, 16).

In recent decades, studies have been published that have conceptualized teaching and learning in education, higher education, and medical education based on a framework. These studies have classified the belief orientation about teaching and learning on a continuum from teaching-centered to learning-centered [15, 16, 17, 18, 19, 12, 9, 3, 20, 21, 22, 23, 24, 25]. One of these frameworks proposed for the context of medical education is the framework of Ottenhoff- de Jonge et al., [22]. They developed a new framework for the context of medical education based on the Samuelowicz and Bain framework [12], which focused on medical educators' beliefs about teaching, learning, and knowledge. Their proposed framework is comprised of a two-dimensional matrix in belief orientations (including six belief orientations: imparting information, transmitting structured knowledge, facilitating understanding, helping the student develop expertise, sharing the responsibility for developing expertise, and negotiating meaning) and nine belief dimensions (including desired learning outcomes, expected use of knowledge, responsibility for transforming knowledge, nature of knowledge, students' existing conceptions, teacher-student interaction, creation of a conducive educational environment, professional development and student motivation). We selected this framework for the following three reasons. First, this framework was largely matched with the subject of our research and therefore had the best fit. Second, this framework is the most comprehensive of all the frameworks developed in the literature. Third, this framework has been developed for the context of medical education.**]**

**[Setting and participants**

This study, including a wide variety of participants, focuses on the clinical environment of undergraduate medical education (UME).  Different participants explained a wide range of perspectives regarding the research topic. In addition, we purposefully selected both groups of experienced clinical teachers and medical education specialists (educational specialists not involved in clinical practice) to address potential differences in beliefs about teaching and learning that may result from engaging in clinical practice. In general, experienced clinical teachers, medical education specialists, and students were present in the second sub-study. Thirty-eight people were recruited for the second sub-study, and 31 participated in web-based interviews. Of these thirty-one people, twenty-two were fifth, sixth, and seventh-year students practically engaged in clinical education (internship), and nine were medical education specialists and clinical teachers. The students were from the Tehran and Shahid Beheshti Universities of Medical Sciences. Fourteen students were male, and eight were female. Finally, telephone interviews were conducted with seven medical education specialists and clinical teachers. Of the sixteen medical education experts and clinical teachers participating in web-based and telephone interviews, nine were male, and seven were female. The participants of the third sub-study were ten people who were organized as an expert panel. Six were medical education specialists (three male and three female), and four experienced clinical teachers (three male and one female). The clinical professors and medical education specialists who participated in this research were from Tehran, Shahid Beheshti, Shiraz, Guilan, and Ahvaz Jondishapur Universities of medical sciences. The Inclusion criteria for medical education specialists included having a Ph.D. in medical education from Iran or abroad, being a faculty member with academic rank (Assistant professor, Associate professor, and Full professor), and at least ten years of work experience in the field and practice of medical education. The inclusion criteria for clinical teachers included expertise in one of the clinical disciplines, faculty membership, at least fifteen years of teaching experience, and academic rank (Assistant Professor, Associate Professor, and Full Professor). We developed these criteria to locate faculty members whose knowledge and experiences would be enlightening.**]**

**[Design of the study**

While mixed method research design refers to combining at least one quantitative and one qualitative method, the multi-method research design approach is not limited solely (and entirely) to combining quantitative and qualitative methods. Multi-method research design integrates several quantitative methods or several qualitative methods, or a combination of both methods (26) to make a richer understanding about the subject under study. Tashakkori and Teddlie (27) define the multi-method research as a type of research design in which multiple methods or worldviews are utilized to collect data. This research comprised three sub-studies that conducted using several methods (each of the methods performed rigorously and complete in itself, in one project) the results were then triangulated to form a complete whole.**]**

**[**

Formulating the research question

Conducting a systematic search

Screening and selecting appropriate qualitative studies

Critical appraisal of studies and extracting the required data from included studies

Analyzing and synthesizing of findings of qualitative studies

Maintaining quality control (Trustworthiness)

Presenting results (conceptual framework)

**Figure 1. The seven-step method of meta-synthesis**]

**[**In other words, if the research question is well-formulated in review studies, it can guide the determination of eligibility criteria, the search for studies, the collection of data from included studies, and the presentation of findings. PICO is the most common structure used to define a researchable question. Health researchers and professionals extensively employ it to develop searchable questions which provide relevant and accurate results. The PICO strategy ensures that the research question drives the entire review process.

The PICO framework requires the researcher to design the research question based on the following four elements (29):

P: Population (Patient or the problem to be addressed)

I: Intervention (Exposure to be considered–treatments/tests)

C: Control (Control or comparison intervention treatment/placebo/standard of care)

O: Outcome (Outcome of interest)

As the PICO tool does not currently accommodate terms related to qualitative research or specific qualitative designs, this framework for searching and synthesizing qualitative evidence has been modified to PICOS, where the "S" refers to the study design (30), thus limiting the number of irrelevant articles.**]**

**[**Study screening was performed based on inclusion and exclusion criteria by two authors (Hamed Khani and Soleiman Ahmady).**]**

**[**To ensure the data's trustworthiness, Maxwell [31, 32] criteria, such as descriptive, Interpretive, and theoretical validity, as well as Kvale [33] criteria, such as pragmatic validity, were considered in this sub-study.

Descriptive validity refers to the factual accuracy of data (31, 32). In meta-synthesis studies, this type of validation means identifying all relevant research reports and a detailed description of each study's report (28). Utilizing all channels to search for studies, consulting with librarians and information science experts, contacting the authors of the studies included in the meta-synthesis to resolve any possible ambiguities, utilizing reference management software, and maintaining the audit trail or documentation of all research steps and processes were employed to increase descriptive validity. Interpretive validity is the complete and fair representation of the meanings attributed to the phenomenon under study by the participants. (31, 32). In meta-synthesis studies, the actors of the study are the researchers whose study report is included in your project. They are not the participants who were the study subjects (28). It is necessary to hold regular meetings to increase the interpretive validity of strategies, such as meetings to discuss the evaluation strategies of the study report. The critical evaluation of the study report under the supervision of the supervisor, two internal and external reviewers, and two peer students, contacting the authors of the studies included in the meta-synthesis to resolve any potential ambiguities, and maintaining the audit trail or documentation of all research steps and processes were employed.

Theoretical validity refers to the theoretical constructs the researcher deals with during the study (31, 32). The primary data in a meta-synthesis study consist of the findings of the studies included in your project. Accordingly, theoretical validity in meta-synthesis studies depends on the credibility of the researcher's methods to develop the integration and researcher interpretation of the researchers' findings of the included studies. To increase the theoretical validity, strategies such as consulting with the supervisor and a number of experts in qualitative methodology and meta-synthesis, as well as maintaining the audit trail or documenting all research steps and processes, were employed.

Pragmatic validity refers to the utility and transferability of knowledge (33). In meta-synthesis studies, pragmatic validity refers to applicability, timeliness, and translatability to synthesizing evidence and the researcher's product (28). Strategies such as expert peer review, maintaining the audit trail, and documenting all research procedures were implemented to increase the pragmatic validity.**]**

**[**five main open-ended questions (Supporting Information file 1). These questions were formulated based on Clark's situation analysis literature and theoretical foundations. In other words, to develop these questions, situational, social worlds/arenas, and positional maps were used as the main strategies of situational analysis. In addition, the analyzed content of documents and curriculum were also used to create these questions. The supervisor, two qualitative research experts, and two peer students evaluated the validity and relevance of the questions. The necessary changes were made based on their feedback, and the final questions were developed.**]**

**[**All methods were conducted in accordance with the ethical principles of the Declaration of Helsinki. The Declaration of Helsinki is one of the most important international documents on ethics in research. The World Medical Association (WMA) has developed the Declaration of Helsinki as a statement of ethical principles for medical research involving human subjects, including research on identifiable human materials and data. The first version of this declaration was adopted by the 18th Assembly of the World Medical Association in Helsinki, Finland, in June 1964. Since then, it has been revised seven times, with the most recent update occurring in 2013 (41). Subject classification and clauses of the latest version of this declaration (2013) include 12 subjects and 37 clauses. In general, the basic principles of this declaration include which protection of life, health, dignity, integrity, right to self-determination, privacy, and confidentiality of personal information of research subjects is the duty of physicians and researchers involved in medical research (41, 42).**]**

**[Discussion**

Based on previous research, learning-centered and teaching or content-centered orientations are separate categories (45) and have their characteristics. However, the results of other studies indicate that these two orientations are poles of a continuum (46, 12, 22). This view defines the teaching or content-centered orientation as "an approach that is not learning-centered. However, the result of our research combines the two opposing viewpoints presented previously. On the one hand, it provides a continuum with three orientations: teaching-centered, learning-centered, and teaching-learning-centered.

On the other hand, in the presented framework, the seven dimensions, such as; pedagogical, context, content, budget, educational leadership, social, and technology, have their characteristics according to the belief orientation, which has intensified the boundary between these orientations. For example, the nature of the social dimension introduced in other research (47, 12, 22) under the title of student-teacher interaction is different in these three orientations. In teaching-centered orientation, the nature of social dimension and interaction is one-sided by the teacher. In a learning-centered orientation, its nature is mutual to maintain students' attention and focus. In teaching-learning-centered orientation, its nature is network and interactional to negotiate and build a collaborative community. In other words, contrary to the research of Kember & Kwan (47) and consistent with the research of Samuelowicz & Bain (12), Postareff & Lindblom-Ylanne (20), and Ottenhoff-de Jonge et al. s(22), the boundary between the three orientations presented in our research is determined by the nature of the social dimension and interaction, not the interaction and the social dimension per se.

In contrast to what Samuelowicz and Bain (12) assert in their study, the line between teaching-centered and learning-centered orientations is relatively "hard." In our study, however, and following the framework of Ottenhoff-de Jonge et al. (22), the unique characteristics of each orientation's seven dimensions highlight the difference between teaching-centered, learning-centered, and teaching-learning-centered orientations(see Table 3).

Regarding the belief orientation toward teaching, previous research has identified two approaches, teaching-centered (content-focused) and learning-centered (47, 12, 20, 22, 16). But the present study's findings suggest that the theory of teaching approaches should transcend the dichotomy between learning-centered and teaching-centered approaches. And in medical education, to improve teaching quality, the relationship between these two approaches should be emphasized; as a consequence of this complex relationship, the teaching-learning-centered approach emerges. Theoretically, teaching-learning orientation means teachers should apply the principles and rules of different learning theories, such as constructivism, behaviorism, and cognitivism, to teach effectively. In other words, based on this orientation, educators should reconcile different learning theories such as behaviorism, cognitive, and constructivism in teaching practice.**]**

**[**While there may be valid articles, studies, and frameworks in the literature**]**

**The changes to the manuscript are shown in track changes.**

**Reviewer 2**

**Reviewer's comment:** Thank you for the opportunity to review this interesting manuscript. Although I am positive with the study, I still have some comments for improvement.

**Authors’ response:** we greatly appreciate your kind and motivating words. We appreciate the time and effort that you dedicated to providing feedback on our manuscript and are grateful for the insightful comments on and valuable improvements to our paper.

**Reviewer's comment:** First, the introduction needs to highlight novelty offered in the study. This can be done by reviewing more updated research on the topic.

**Authors’ response:** Thank you for your valuable suggestion. We have addressed this concern as suggested by you.

**The revised text reads as follows:**

**[**Teachers' belief orientation about teaching and learning should be considered to develop the framework of effective educational behaviors. "Beliefs" refer to perceptions and conceptions about teaching and learning, which are formed throughout life, deeply rooted, and contain cognitive and affective aspects (7, 8). The body of research shows that epistemological beliefs are one of the driving forces behind educators' educational behaviors and their choices in teaching design and delivery. In other words, how teachers teach and even teaching practice is unconsciously influenced by their conceptions and perceptions of teaching and learning (9, 10, 11, 12, 13, 14, 15, 16).

In recent decades, studies have been published that have conceptualized teaching and learning in education, higher education, and medical education based on a framework. These studies have classified the belief orientation about teaching and learning on a continuum from teaching-centered to learning-centered [15, 16, 17, 18, 19, 12, 9, 3, 20, 21, 22, 23, 24, 25]. One of these frameworks proposed for the context of medical education is the framework of Ottenhoff- de Jonge et al., [22]. They developed a new framework for the context of medical education based on the Samuelowicz and Bain framework [12], which focused on medical educators' beliefs about teaching, learning, and knowledge. Their proposed framework is comprised of a two-dimensional matrix in belief orientations (including six belief orientations: imparting information, transmitting structured knowledge, facilitating understanding, helping the student develop expertise, sharing the responsibility for developing expertise, and negotiating meaning) and nine belief dimensions (including desired learning outcomes, expected use of knowledge, responsibility for transforming knowledge, nature of knowledge, students' existing conceptions, teacher-student interaction, creation of a conducive educational environment, professional development and student motivation). We selected this framework for the following three reasons. First, this framework was largely matched with the subject of our research and therefore had the best fit. Second, this framework is the most comprehensive of all the frameworks developed in the literature. Third, this framework has been developed for the context of medical education.**]**

**The changes to the manuscript are shown in track changes.**

**Reviewer's comment:** Second, the findings should be discussed based on both theoretical perspectives and empirical evidence. I suggest the authors review recent wok on the topic.

**Authors’ response:** We agree with your comment. Thank you for your valuable recommendation.

We have addressed this concern as suggested by you. We have used recent studies to formulate the discussion of our article.

**The revised text reads as follows:**

**[**Based on previous research, learning-centered and teaching or content-centered orientations are separate categories (45) and have their characteristics. However, the results of other studies indicate that these two orientations are poles of a continuum (46, 12, 22). This view defines the teaching or content-centered orientation as "an approach that is not learning-centered. However, the result of our research combines the two opposing viewpoints presented previously. On the one hand, it provides a continuum with three orientations: teaching-centered, learning-centered, and teaching-learning-centered.

On the other hand, in the presented framework, the seven dimensions, such as; pedagogical, context, content, budget, educational leadership, social, and technology, have their characteristics according to the belief orientation, which has intensified the boundary between these orientations. For example, the nature of the social dimension introduced in other research (47, 12, 22) under the title of student-teacher interaction is different in these three orientations. In teaching-centered orientation, the nature of social dimension and interaction is one-sided by the teacher. In a learning-centered orientation, its nature is mutual to maintain students' attention and focus. In teaching-learning-centered orientation, its nature is network and interactional to negotiate and build a collaborative community. In other words, contrary to the research of Kember & Kwan (47) and consistent with the research of Samuelowicz & Bain (12), Postareff & Lindblom-Ylanne (20), and Ottenhoff-de Jonge et al. s(22), the boundary between the three orientations presented in our research is determined by the nature of the social dimension and interaction, not the interaction and the social dimension per se.

In contrast to what Samuelowicz and Bain (12) assert in their study, the line between teaching-centered and learning-centered orientations is relatively "hard." In our study, however, and following the framework of Ottenhoff-de Jonge et al. (22), the unique characteristics of each orientation's seven dimensions highlight the difference between teaching-centered, learning-centered, and teaching-learning-centered orientations(see Table 3).

Regarding the belief orientation toward teaching, previous research has identified two approaches, teaching-centered (content-focused) and learning-centered (47, 12, 20, 22, 16). But the present study's findings suggest that the theory of teaching approaches should transcend the dichotomy between learning-centered and teaching-centered approaches. And in medical education, to improve teaching quality, the relationship between these two approaches should be emphasized; as a consequence of this complex relationship, the teaching-learning-centered approach emerges. Theoretically, teaching-learning orientation means teachers should apply the principles and rules of different learning theories, such as constructivism, behaviorism, and cognitivism, to teach effectively. In other words, based on this orientation, educators should reconcile different learning theories such as behaviorism, cognitive, and constructivism in teaching practice.**]**

**The changes to the manuscript are shown in track changes.**

**Reviewer's comment:** Lastly, I noted some grammatical errors and typos in the manuscript. Please check them and have the manuscript proofed professionally.

**Authors’ response:** Thank you for your feedback and for your careful attention to detail. We have addressed this concern as suggested by you and the manuscript has been re-edited by one expert English editor and we have uploaded the Certificate of English Language Editing.
